# Comparisons of cardiovascular dysautonomia and cognitive impairment between de novo Parkinson’s disease and de novo dementia with Lewy bodies

**DOI:** 10.1186/s12883-020-01928-5

**Published:** 2020-09-18

**Authors:** Hisayoshi Oka, Tadashi Umehara, Atsuo Nakahara, Hiromasa Matsuno

**Affiliations:** 1 Department of Neurology, Daisan Hospital, The Jikei University School of Medicine, 4-11-1 Izumihoncho, Komae-shi, Tokyo, 201-8601 Japan; 2 Department of Neurology, The Jikei University School of Medicine, 3-19-18, Tokyo, 105-8471 Japan

**Keywords:** Orthostatic hypotension, Daily blood pressure fluctuation, Cognitive decline, Parkinson’s disease, Dementia with Lewy bodies

## Abstract

**Background:**

Cognitive impairment may be correlated with cardiovascular dysautonomia, including blood pressure (BP) dysregulation, in Parkinson’s disease (PD), but the association between these factors in dementia with Lewy bodies (DLB) is uncertain. This study aimed to clarify whether cardiovascular dysautonomia had an influence on cognitive function in Lewy body disease or not.

**Methods:**

Ninty-nine patients with de novo PD (*n* = 75) and DLB (*n* = 24) were evaluated using the Mini-Mental State Examination (MMSE) and Frontal Assessment Battery (FAB). Cardiac ^123^I-metaiodobenzylguanidine (MIBG) scintigraphy, orthostatic hypotension (OH), supine hypertension (SH), postprandial hypotension (PPH), nocturnal BP fall in 24-h ambulatory blood pressure monitoring (ABPM) and constipation were estimated. Associations of these factors with cognitive and executive dysfunction were examined.

**Results:**

In DLB, MIBG uptake was reduced and OH, PPH and SH were severely disturbed, compared to PD. The nocturnal BP fall in ABPM was lower in DLB, and the failure of nocturnal BP fall in PD was associated with MMSE, after adjustment for other clinical features. FAB was significantly associated nocturnal BP fall, age and SH in PD, but no significant correlations among factors were found for DLB.

**Conclusion:**

The significant association between nocturnal BP dysregulation and cognitive or executive decline in PD might be due to impaired microvascular circulation or invasion of α-synuclein in the CNS. The lack of a correlation of BP insufficiency with cognitive impairment in DLB suggests initial involvement of Lewy body pathology in the neocortex, regardless of Lewy body invasion of the autonomic nervous system.

## Background

Parkinson’s disease (PD) is commonly accepted to be associated with motor symptoms and various non-motor symptoms, including behavioral changes such as depression, sleep disturbance, fatigue and autonomic dysfunction. Autonomic impairment associated with PD is characterized by clinical features of constipation, sweating, orthostatic hypotension (OH) and postprandial hypotension (PPH), even in the early phase [1]. OH occurs through sympathetic noradrenergic dysfunction and is clinically important in 20–50% of patients with PD [1]. Survival depends on the OH status, with a greater risk of death in PD with OH than in PD without OH [2]. OH may also affect cognition [3], daily activities, and quality of life [4]. Patients with PD with OH have significantly worse sustained attention and visual episodic memory [5] and significantly lower scores on the Mini-Mental State Examination (MMSE) [6, 7].

Abnormal blood pressure (BP) fluctuations are also common in PD [8, 9]. The normal nocturnal BP fall in a healthy person disappears in PD, especially in a case with autonomic dysfunction [10, 11]. Cognitive decline in older people is associated with abnormal BP fluctuations, such as the absence of a normal nocturnal BP fall [11, 12], and cognitive impairment in PD has also been associated with abnormal BP fluctuation [13] (Tanaka et al.*,* 2018). Autonomic dysfunction in dementia with Lewy bodies (DLB) is generally more severe than that in PD [14]. The severity of autonomic failure in DLB is intermediate between those in PD and multiple system atrophy [15]. OH is likely to be severer in DLB than in PD because it is thought to be due to Lewy body involvement in the rostral ventrolateral medulla and medullary raphe, which may control sympathetic outflow [15]. However, it is not clear whether cognitive dysfunction in DLB is correlated with blood circulatory insufficiency such as OH and abnormal BP fluctuation, as occurs in PD.

The aims of this study were to examine the association of cardiovascular dysautonomia and cognitive impairments in de novo PD and DLB using ^123^I-metaiodobenzylguanidine (MIBG) uptake by the heart, responses of BP and plasma norepinephrine in a head-up tilt-table test (HUT), a 75-g oral glucose tolerance test (75-g OGTT) for PPH, 24-h ambulatory blood pressure monitoring (ABPM) and constipation.

## Methods

### Participants

The subjects were 75 de novo PD patients who were diagnosed using the criteria for PD proposed by the UK Parkinson’s Disease Society Brain Bank [16], and 24 de novo DLB patients with a central feature and two or more core features in diagnostic criteria, which is sufficient for diagnosis of probable DLB [17]. All patients were examined at Disan Hospital, The Jikei University School of Medicine, between January 2012 and March 2018, and were diagnosed by at least two neurologists. We also used a 1-year rule to distinguish DLB from PD with dementia [17].

Patients with overt diabetes or clinically relevant cardiac disease, and those who had been treated with tricyclic antidepressants, tetracyclic antidepressants, serotonin reuptake inhibitors, and serotonin and norepinephrine reuptake inhibitors were excluded from the study. None of the patients had received levodopa, other anti-Parkinson drugs, or treatment for OH. Global cognition and executive function were evaluated using the MMSE and the Frontal Assessment Battery (FAB). No patient had atrophy on brain MRI of the putamen, brainstem or cerebellum. If patients were already receiving antihypertensive drugs, such drugs were withdrawn at least 48 h before evaluation of OH. All patients received levodopa or a dopamine agonist for their parkinsonism after this study, and all PD patients had a good response.

The motor severity of PD was assessed using the Unified Parkinson’s Disease Rating Scale (UPDRS) motor score. The patients were divided into two subgroups, akinetic-rigid and tremor-dominant or mixed type based on the tremor and non-tremor scores, which were obtained using part III of the UPDRS [18].

### Study design

Cardiac sympathetic denervation was evaluated using MIBG scintigraphy. The ratio of the average pixel count in the heart (H) to that in the mediastinum (M) (H/M ratio) at 15 min (early) and 3 h (delayed) after injection of 111 MBq 123I-MIBG (Fujifilm RI Pharma Co., Ltd. Tokyo, Japan) was calculated, as we have previously reported [19].

We assessed olfactory function using the odor stick identification test Japan (OSIT-J) (Daiichi Yakuhin Sangyo Co. Ltd., Tokyo, Japan), as described in our previous study [20]. The OSIT-J score was defined as the number of correct responses for the 12 odorants. It has been shown to be significantly correlated with those on the University of Pennsylvania Smell Identification Test (UPSIT) and the cross-cultural, smell identification test (CC-SIT) [21, 22].

We performed Head-up tilt-table test (HUT) to all subjects in a silent room maintained at an ambient temperature of 23 to 26 °C, as previously reported in our study [23]. The test was started at 9:00 am after an overnight fast. The subject was tilted to a 60° upright position for 10 min after resting for 20 min in the supine position. Brachial systolic blood pressure (SBP) and diastolic blood pressure (DBP) were measured by an automated sphygmomanometer after 20 min of rest in the supine position and every 1 min after the subject was tilted for up to 10 min. The maximum decreases in SBP and DBP during tilt were evaluated. Plasma norepinephrine concentrations in serum (NE, μg/ml)) were measured with the subjects in a supine position after 20 min of rest and after 10 min in a tilted position. OH was defined as a fall in SBP by ≥20 mmHg [24].

Supine hypertension (SH) was defined as SBP > 140 mmHg or DBP > 90 mmHg, as measured after 20 min of rest in the supine position [25]. Neurogenic SH was defined as a case of OH with SBP > 140 mmHg or DBP > 90 mmHg in the supine position [26].

24-h ambulatory blood pressure monitoring (ABPM) was performed using a noninvasive automated portable recorder in hospitalized patients and outpatients. BP was measured every 30 min during the day (7:00–21:00) and every hour at night (22:00–6:00). SBP was used as an indicator of BP. The nocturnal fall in BP was calculated as: SBP_day_ – SBP_night_/SBP _day_ × 100 (%), where SBP_day_ is the mean SBP during the day, and SBP_night_ is the mean SBP at night. Cases with nocturnal falls in BP of ≥10% (respectively < 10%, no fall) were defined as dipper type (respectively non-dipper, riser), as described in previous studies [27, 28].

We performed 75-g oral glucose tolerance test for evaluation of postprandial hypotension, as described in our previous studies [23, 29]. The 75-g OGTT was started between 9:00 and 10:00 am after overnight fasting (except for non-caloric liquids) in a quiet room at 23 to 26°. We performed HUT followed by the 75-g OGTT on the same day. It was performed on the day after HUT in the instances where it was not possible. The subjects took 75 g of glucose water (calorie content, 300 kcal) after 20 min resting in the supine position, and remained resting and awake in the supine position for 120 min. Brachial SBP and DBP were measured by an automated sphygmomanometer in the supine position after 20 min and every 10 min for the next 120 min,. The maximum drop in SBP was measured during 75-g OGTT test. Postprandial hypotension **(**PPH) was defined as a maximum decrease in SBP of 20 mmHg within 2 h after glucose intake. Constipation was evaluated as the absence of daily defecation, or use of drugs to treat constipation, as described in our previous study [30].

This study was approved by the Ethics Committee of The Jikei University School of Medicine, and all subjects gave written informed consent before enrollment.

### Statistical analysis

Statistical analyses were performed with statistical software (Esumi Co., Ltd., Tokyo, Japan). Differences between groups were compared by Wilcoxon rank sum test for continuous variables. Pairwise comparisons were made using χ2 tests for binary variables. Lepage analysis reported by Marozzi [31] was used to evaluate differences in nocturnal fall in BP. Associations of MMSE and FAB scores with clinical factors such as age, gender, symptom duration, UPDRS motor score, motor subtype, olfaction, cardiac MIBG uptake, BP fall in HUT, NE at rest in the supine position in HUT, nocturnal fall in BP in ABPM, PPH, SH and constipation were evaluated by multiple regression analysis. *P* < 0.05 was considered to indicate significance.

## Results

### Cognitive impairment and cardiovascular dysautonomia in PD and DLB

A comparison of the characteristics of the PD and DLB patients is given in Table 1. DLB patients were older than PD patients, but there was no significant difference in the duration of PD and DLB. PD patients were significantly more frequently female, while DLB patients were more commonly male. There were no significant differences in UPDRS motor scores and motor subtype between PD and DLB cases. DLB patients had more severely impaired olfaction, lower MMSE and FAB scores, a lower H/M ratio in cardiac MIBG uptake, a lower BP fall in HUT, lower NE in a resting supine position in HUT, and a higher prevalence of OH, compared to PD cases. There was a reduced nocturnal fall in BP in ABPM and a higher percentage of non-dipper/riser types among DLB cases. BP fall in PPH and the prevalence of PPH were larger in DLB cases. The prevalence of SH did not differ significantly, but that of neurogenic SH was higher in DLB patients. The prevalence of constipation in DLB cases was also higher than that in PD cases.

**Table 1:** Comparison of clinical characteristics in patients with de novo DLB and PD

Item	PD (*N* = 75)	DLB (*N* = 24)	*p* value ^a^
Age (years)	72.2 ± 9.4	80.0 ± 5.0	0.001
Gender (male/female)	27/48	18/6	0.001
Symptom duration (years)	1.6 ± 1.6	1.9 ± 1.7	0.296
UPDRS motor score	20.3 ± 10.9	21.3 ± 10.8	0.236
Subtype (akinetic-rigid/tremor-dominant or mixed)	45/30	19/5	0.087
Olfaction	4.1 ± 2.8	2.4 ± 2.2	0.010
Mini-mental state examination	27.7 ± 2.2	20.0 ± 4.7	0.001
Frontal assessment battery	15.1 ± 2.8	10.2 ± 3.4	0.001
MIBG H/M ratio delay	1.44 ± 0.33	1.22 ± 0.39	0.001
BP fall in head-up tilt-table test (HUT) (mmHg)	23.6 ± 20.0	48.5 ± 23.6	0.001
Prevalence of OH (−/+)	36/39	1/23	0.001
Norepinephrine at rest in supine position in HUT (μg/ml)	217.9 ± 88.2	172.8 ± 82.5	0.009
Nocturnal fall in BP in ABPM (%)	8.5 ± 9.4	−4.4 ± 12.6	0.001
ABPM type (dipper/non dipper or riser type)	37/38	4/20	0.005
BP fall in postprandial hypotension (mmHg)	18.4 ± 13.1	32.8 ± 17.2	0.001
Postprandial hypotension (−/+)	42/33	6/18	0.008
BP in supine hypertension (mmHg)	134.8 ± 21.5	147.5 ± 19.2	0.011
Supine hypertension (−/+)	45/30	10/14	0.116
Neurogenic supine hypertension (−/+)	56/19	12/12	0.013
Constipation (−/+)	25/50	1/23	0.008

^a^ Wilcoxon rank sum test and Lepage analysis for continuous variables and χ^2^ test for binary variables

*UPDRS* Unified Parkinson’s Disease Rating Scale, *MIBG* ^123^I-metaiodobenzylguanidine, H/M: ratio of the average pixel count in the heart (H) to that in the mediastinum (M), *ABPM* 24-h ambulatory blood pressure monitoring

### Correlations between cognitive impairment and cardiovascular dysautonomia

In multiple regression analyses, MMSE in PD was significantly associated with nocturnal fall in BP in ABPM (*p* = 0.0275), after adjustment for age, disease duration, UPDRS motor score, motor subtype, olfaction, cardiac MIBG scintigraphy, BP fall in HUT, NE at rest in a supine position in HUT, PPH, SH and constipation (Table 2); and FAB in PD was significantly related with nocturnal fall in BP (*p* = 0.0395), aging (*p* = 0.0076) and SH (*p* = 0.0037) (Table 3). In contrast, neither MMSE nor FAB in DLB was associated with nocturnal fall in BP or any other clinical variable (Tables 4, 5).

**Table 2:** Relationships of MMSE with clinical parameters in PD

Clinical parameter	Parameter Estimate	Standard Error	*p*-value ^a^
Age (years)	−0.053	0.031	0.099
Gender (male/female)	0.260	0.594	0.664
Symptom duration (years)	−0.208	0.162	0.204
UPDRS motor score	−0.017	0.026	0.515
Subtype (akinetic-rigid/tremor-dominant or mixed)	−0.906	0.528	0.091
Olfaction	−0.159	0.101	0.121
MIBG H/M ratio delay	−0.0310	0.837	0.971
BP fall in head-up tilt-table test (HUT) (mmHg)	−0.013	0.013	0.330
Norepinephrine at rest in supine position in HUT (μg/ml)	0.005	0.003	0.095
Nocturnal fall in BP in ABPM (%)	0.066	0.029	0.028
BP fall in postprandial hypotension (mmHg)	0.028	0.020	0.169
BP in supine hypertension (mmHg)	−0.180	0.013	0.165
Constipation	−0.018	0.622	0.519

^a^ Analysis were performed by multiple regression analysis

Abbreviations are as shown in Table 1

**Table 3:** Relationships of FAB with clinical parameter in PD

Clinical parameter	Parameter Estimate	Standard Error	*p*-value ^a^
Age (years)	− 0.098	0.036	0.008
Gender (male/female)	−0.867	0.667	0.200
Symptom duration (years)	−0.028	0.183	0.880
UPDRS motor score	−0.012	0.029	0.686
Subtype (akinetic-rigid/tremor-dominant or mixed)	−0.551	0.595	0.358
Olfaction	−0.002	0.114	0.990
MIBG H/M ratio delay	−0.118	0.944	0.901
BP fall in head-up tilt-table test (HUT) (mmHg)	0.018	0.015	0.225
Norepinephrine at rest in supine position in HUT (μg/ml)	0.002	0.003	0.650
Nocturnal fall in BP in ABPM (%)	0.070	0.033	0.040
BP fall in postprandial hypotension (mmHg)	0.002	0.022	0.920
BP in supine hypertension (mmHg)	−0.043	0.014	0.004
Constipation	−0.199	0.701	0.778

^a^ Analysis were performed by multiple regression analysis

Abbreviations are as shown in Table 1

**Table 4:** Relationships of MMSE with clinical parameters in DLB

Clinical parameter	Parameter Estimate	Standard Error	*p*-value ^a^
Age (years)	−0.435	0.255	0.118
Gender (male/female)	−3.063	3.454	0.396
Symptom duration (years)	0.959	0.807	0.262
UPDRS motor score	−0.176	0.128	0.198
Subtype (akinetic-rigid/tremor-dominant or mixed)	1.492	3.664	0.693
Olfaction	0.566	0.651	0.405
MIBG H/M ratio delay	1.136	3.243	0.733
BP fall in head-up tilt-table test (HUT) (mmHg)	0.039	0.049	0.448
Norepinephrine at rest in supine position in HUT (μg/ml)	−0.024	0.019	0.245
Nocturnal fall in BP in ABPM (%)	0.095	0.143	0.524
BP fall in postprandial hypotension (mmHg)	0.110	0.100	0.298
BP in supine hypertension (mmHg)	−0.010	0.072	0.895
Constipation	−6.900	5.725	0.256

^a^ Analysis were performed by multiple regression analysis

Abbreviations are as shown in Table 1

**Table 5:** Relationships of FAB with clinical parameters in DLB

Clinical parameter	Parameter Estimate	Standard Error	*p*-value ^a^
Age (years)	−0.326	0.188	0.114
Gender (male/female)	−1.791	2.551	0.499
Symptom duration (years)	0.221	0.596	0.719
UPDRS motor score	−0.169	0.094	0.104
Subtype (akinetic-rigid/tremor-dominant or mixed)	0.081	2.706	0.976
Olfaction	−0.177	0.481	0.721
MIBG H/M ratio delay	1.731	2.395	0.486
BP fall in head-up tilt-table test (HUT) (mmHg)	0.006	0.036	0.868
Norepinephrine at rest in supine position in HUT (μg/ml)	0.003	0.014	0.829
Nocturnal fall in BP in ABPM (%)	0.016	0.106	0.885
BP fall in postprandial hypotension (mmHg)	−0.016	0.074	0.830
BP in supine hypertension (mmHg)	−0.009	0.053	0.875
Constipation	1.464	4.227	0.736

^a^ Analysis were performed by multiple regression analysis

Abbreviations are as shown in Table 1

## Discussion

In this study, DLB patients clearly had more severe cognitive decline compared with PD patients. Olfaction was more impaired in DLB than PD. Olfactory dysfunction has recognized in PD and has been associated with cognitive dysfunction in PD [20, 32]. De novo PD with mild cognitive decline shows olfactory impairment compared with patients without cognitive dysfunction [33]. This is consistent with DLB patients with impaired cognition having more severe olfactory dysfunction than that in PD patients. Our study has also found that DLB patients were commonly male in contrast with that PD patients were frequently female, and that patients with diffuse or limbic Lewy body pathologies tended to be found in male has been already reported [34].

MIBG uptake in scintigraphy indicating cardiac sympathetic denervation was lower in patients with DLB than in those with PD. BP fall on standing was significantly larger, NE at rest was lower, and the BP fall in PPH and SH were larger in DLB. Neurogenic SH differed significantly between PD and DLB cases, but not the prevalence of SH. The prevalence of constipation in DLB was higher than in PD, which suggests that intestinal autonomic dysfunction might be severer in DLB. Overall, our findings suggest that DLB involves wider spread severer sympathetic and parasympathetic autonomic dysfunction in peripheral and central nervous system compared with PD [14].

The nocturnal fall in BP in ABPM, which was found in healthy persons, was reduced more in DLB than PD. Abnormal daily BP fluctuation in PD [35, 36] has been associated with cardiovascular dysautonomia [36], but has rarely been reported in DLB. PD patients with this condition, including reduced or reverse nocturnal BP fall on ABPM, have also been found to have a higher prevalence of OH [35]. More profound impairment of the nocturnal fall in BP might be found in DLB with severe cardiovascular autonomic dysfunction. It was previously reported that cognitive function has been linked to abnormal BP fluctuation in PD [11, 13], including an abnormality in nocturnal BP fall in ABPM. The novel finding in our study is that nocturnal BP abnormality was associated with cognitive and executive dysfunction in early stage and de novo PD patients after adjustment for other dysautonomia of cardiovascular autonomic factors, including OH, PPH, SH and cardiac sympathetic impairment indicated by MIBG uptake insufficiency.

Del Pino [37] has already mentioned that cardiovascular autonomic dysfunction was associated with cognitive and neuropsychiatric impairment in Lewy body disease, using a number of tests to assess autonomic function such as hemodynamic parameters during deep breathing, the Valsalva maneuver, and head up tilt test. Several pathogeneses have been suggested for the association of cognitive dysfunction and BP abnormality, but the underlying mechanism is still unclear. The Braak hypothesis [38] suggests that Lewy body (LB) pathology initially occurs in the olfactory nucleus and dorsal motor nucleus and progressively ascends through the brain stem to the cortex, causing noradrenergic and dopaminergic neuronal degeneration that results in progression of motor, cognitive, and autonomic impairment. Cognitive decline in PD has been associated with specific patterns of LB density in the entorhinal cortex and anterior cingulate cortex [39], which plays a role in autonomic nervous system (ANS) control, including the higher centers of autonomic regulations [40]. Involvement of the anterior cingulate cortex might simultaneously cause cognitive impairment and cardiovascular sympathetic failure.

Noradrenergic projection from the locus coeruleus (LC) spreads extensively in the whole brain cortex, including the hippocampus, entorhinal and mediotemporal cortex, cingulate gyrus and neocortex. Tyrosine hydroxylase immunoreactivity is lost in neurons projecting from the LC due to the LB pathology in PD [41]. Involvement of the noradrenergic neurons of the LC is increasingly recognized as a potential major contributor to cognitive manifestations in early PD, particularly for impaired attention [42]. The LC also projects to the parasympathetic neurons of the dorsal motor nucleus of the vagus (the nucleus ambiguous), while the descending pathway projects to sympathetic preganglionic neurons in the spinal cord [42] in autonomic nervous system. Therefore, the LC should influence cardiovascular modulation via insufficiency of cardiac parasympathetic and cardiovascular sympathetic function. The LC also regulates part of the wake-promoting circuit with the suprachiasmatic nucleus and dorsomedial hypothalamus [43]. Therefore, spoiling of the LC may cause abnormal daily BP fluctuation such as reduced nocturnal fall in BP, in addition to cardiac parasympathetic and cardiovascular sympathetic failure.

BP insufficiency such as OH, including circadian rhythm failure, is associated with increased white matter hyperintensities (WMHs) on MRI, even in older people [44, 45]. Cognitive scores and WMH volumes on MRI are severely reduced in PD patients with both OH and SH, and lability of BP circulation may be related to cognitive function and WMH volume on MRI [11]. Our study revealed that abnormal BP fluctuation, and especially a reduced nocturnal fall in BP, was associated with cognitive and executive function in PD, after adjustment for other autonomic characteristics, including cardiovascular sympathetic function reflected by cardiac MIBG uptake, OH, PPH, circulatory NE concentration, and constipation. This suggests that increased lability of daily BP and nocturnal BP is a risk factor for cognitive impairment, even in early de novo PD. Furthermore, FAB scores, but not MMSE scores, were correlated with SH and aging in our PD patients. This may indicate executive dysfunction occurred due to damage of prefrontal areas, which is readily attributable to cerebrovascular circulatory insufficiency of the cortex, white matter or age-related changes in the brain in PD [24].

In contrast to PD, we found that cognitive and executive impairments in DLB patients were not correlated with lability of BP and nocturnal BP. Dysautonomia in DLB seems to be severer than that in PD from our results and previous reports [14, 15]. It is uncertain whether PD and DLB including PDD are separate disease entities or parts of the same disease spectrum. LB pathology in PD is restricted to the brainstem and limbic regions, while the pathology more quickly extends to the neocortex in DLB. LB pathology in PD is also not so widely distributed in autonomic nervous organs, compared with that in DLB. The discrepancy between cardiovascular and cognitive dysfunction in DLB might suggest that regional invasion of LB pathology differs between the neocortex and sympathetic autonomic center. Braak’s hypothesis [38] suggests that α synucleinopathy initially involves the intestinal organ and ascends to the brainstem, including the dorsal motor nucleus of the vagus, LC, medullary reticular formation, raphe nuclei, and peripheral sympathetic nervous system. These organs are associated with modulation of cardiovascular autonomic regulation in early PD.

Cognitive dysfunction in PD might occur due to white matter damage from BP dysregulation and noradrenergic decline of the LC [41]. Some papers suggested that cognition dysfunction should be associated with cardiovascular autonomic failure if LB pathology involves the ACC or insular cortex. Cognitive decline has already progressed due to involvement of LB pathology in brain cortex, so that cognitive impairment in DLB should not be strongly influenced by BP dysregulation. Alzheimer disease (AD) pathology with hyperphosphorylated tau and amyloid-β (Aβ) may also contribute to cognitive decline in DLB and PD. Aβ plaques are significantly more common in cortical and subcortical regions in DLB compared to PDD [46, 47], and DLB displays concurrent AD-related pathology compared to PDD [48]. Cardiovascular dysautonomia including reduced cardiac MIBG uptake and OH is not as impaired in AD compared to DLB [49]. Thus, AD pathology in DLB may be not correlated with ANS effects, and the increased AD pathology may induce dissociation between cognitive and BP dysregulation in DLB compared to PD.

## Conclusion

BP dysregulation, especially reduced nocturnal BP fall, was associated with cognitive and executive decline in PD, and this may be driven by impaired microvascular circulation of white matter or infiltration of α synuclein from the peripheral ANS to the CNS, such as the LC or ACC. The absence of a correlation between cognitive and BP dysregulation in DLB is due to earlier spread of LB pathology to the neocortex in early stage compared with PD, while the more severe AD pathology in the cortex in DLB compared to PD might also contribute to dissociation of cognitive dysfunction and BP abnormality. Therefore, treatment for BP dysregulation may prevent progression of cognitive decline in PD, but not in DLB.

The current study has a strength and limitation. Enrolled PD and DLB patients are newly diagnosed who did not take any dopaminergic medication. The association between cognitive impairment and autonomic dysfunctions has not interfered by results from antiparkinsonian treatment. A limitation of the study is that the number of DLB patients is relatively small compared to PD patients. This may explain why the correlation between cognitive impairment and BP abnormality in PD was not found in DLB. However, it is speculated that earlier spread of LB pathology to the neocortex in early stage and the more severe AD pathology in the cortex in DLB compared to PD could also be a factor that produced no correlation between cognitive dysfunction and BP abnormality.

## Data Availability

The datasets used and/or analyzed during the current study are available from the corresponding author on reasonable request.
